# Clinical validation of an in-house quantitative real time PCR assay for cytomegalovirus infection using the 1^st^ WHO International Standard in kidney transplant patients

**DOI:** 10.1590/2175-8239-JBN-2020-0214

**Published:** 2021-05-10

**Authors:** Cassia F.B. Caurio, Odelta S. Allende, Roger Kist, Kênya L. Santos, Izadora C.S. Vasconcellos, Franciéli P. Rozales, Daiane F. Dalla Lana, Bruno M. Praetzel, Ana Paula Alegretti, Alessandro C. Pasqualotto

**Affiliations:** 1 Santa Casa de Misericórdia de Porto Alegre, Porto Alegre, RS, Brasil.; 2 Universidade Federal de Ciências da Saúde de Porto Alegre, Porto Alegre, RS, Brasil.; 3 Hospital de Clínicas de Porto Alegre, Porto Alegre, RS, Brasil.

**Keywords:** Cytomegalovirus, PCR, Drug Therapy, Diagnosis, Citomegalovírus, PCR, Tratamento Farmacológico, diagnóstico

## Abstract

**Introduction:**

Cytomegalovirus (CMV) is one of the most common agents of infection in solid organ transplant patients, with significant morbidity and mortality.

**Objective:**

This study aimed to establish a threshold for initiation of preemptive treatment. In addition, the study compared the performance of antigenemia with qPCR results.

**Study design:**

This was a prospective cohort study conducted in 2017 in a single kidney transplant center in Brazil. Clinical validation was performed by comparing in-house qPCR results, against standard of care at that time (Pp65 CMV Antigenemia). ROC curve analysis was performed to determine the ideal threshold for initiation of preemptive therapy based on the qPCR test results.

**Results:**

Two hundred and thirty two samples from 30 patients were tested with both antigenemia and qPCR, from which 163 (70.26%) were concordant (Kappa coefficient: 0.435, *p*<0.001; Spearman correlation: 0.663). PCR allowed for early diagnoses. The median number of days for the first positive result was 50 (range, 24-105) for antigenemia and 42 (range, 24-74) for qPCR (*p*<0.001). ROC curve analysis revealed that at a threshold of 3,430 IU/mL (Log 3.54), qPCR had a sensitivity of 97.06% and a specificity of 74.24% (AUC 0.92617 ± 0.0185, *p*<0.001), in the prediction of 10 cells/10^5^ leukocytes by antigenemia and physician's decision to treat.

**Conclusions:**

CMV Pp65 antigenemia and CMV qPCR showed fair agreement and a moderate correlation in this study. The in-house qPCR was revealed to be an accurate method to determine CMV DNAemia in kidney transplant patients, resulting in positive results weeks before antigenemia.

## Resumo

**Introdução:**

Citomegalovírus (CMV) é um dos agentes infecciosos mais comuns em pacientes com transplante de órgãos sólidos, com morbidade e mortalidade significativas.

**Objetivo:**

Este estudo visou estabelecer um limite para o início do tratamento preemptivo. Além disso, comparou o desempenho da antigenemia com os resultados da qPCR *in house.*

**Desenho do estudo:**

Este foi um estudo de coorte prospectivo realizado em 2017 em um centro único de transplante renal no Brasil. A validação clínica foi realizada comparando resultados de qPCR *in house*, com o padrão de atendimento na época (Antigenemia para CMV Pp65). A análise da curva ROC foi realizada para determinar o limite ideal para o início da terapia preemptiva baseado nos resultados do teste qPCR *in house*.

**Resultados:**

232 amostras de 30 pacientes foram testadas com antigenemia e qPCR, das quais 163 (70,26%) foram concordantes (Coeficiente Kappa: 0,435, *p*<0,001; Correlação Spearman: 0,663). PCR permitiu diagnósticos precoces. O número médio de dias para o primeiro resultado positivo foi 50 (intervalo, 24-105) para antigenemia e 42 (intervalo, 24-74) para qPCR (*p*<0,001). A análise da curva ROC revelou que em um limite de 3.430 UI/mL (Log 3,54), qPCR teve sensibilidade de 97,06% e especificidade de 74,24% (AUC 0,92617 ± 0,0185, *p*<0,001), na previsão de 10 células/10^(5)^ leucócitos por antigenemia e na decisão do médico de tratar.

**Conclusões:**

Antigenemia para CMV Pp65 e qPCR para CMV mostraram uma concordância aceitável e uma correlação moderada neste estudo. qPCR *in house* revelou-se um método preciso para determinar DNAemia do CMV em pacientes transplantados renais, obtendo resultados positivos semanas antes da antigenemia.

## Background

Cytomegalovirus (CMV) (Order *Herpesvirales*, Family *Herpesviridae*, Subfamily *Betaherpesvirinae*, Genus *Cytomegalovirus*, Species *Human betaherpesvirus 5*) is one of the most relevant causes of infection in transplant organ recipients, resulting in significant morbidity and mortality^1^. Infection can originate from the transplanted organ or more commonly due to reactivation of previous (latent) CMV infection in the transplant recipient^2^.

Most patients at risk of CMV infection/disease are monitored with diagnostic tests aiming for an early detection of CMV infection, in the so called 'preemptive' strategy. Laboratory monitoring for preemptive therapy was performed in early years with Pp65 CMV antigenemia. However, molecular assays have replaced antigenemia to become the gold-standard for CMV^3^ diagnosis and monitoring. However, due to large inter-assay variations, no universal consensus has been reached on the threshold to initiate therapy against CMV^3^^-^ 5.

In this scenario, this study aimed to establish a threshold for initiation of preemptive treatment against CMV in a cohort of kidney transplant patients in Brazil. In addition, the study compared the performance of antigenemia and a novel in-house quantitative real time PCR (qPCR) assay, which was calibrated using the 1^st^ WHO International Standard for Human CMV.

## Material and methods

### Patients and samples

This was a prospective observational cohort study conducted between January and April 2017. All adult (older than 18 years old) kidney transplant patients being taking care at Santa Casa de Misericórdia de Porto Alegre, Brazil, were considered for inclusion in the study. Patients were followed weekly for at least three months after kidney transplantation. Plasma samples for CMV qPCR tests were collected weekly using 4 mL EDTA tubes. Samples were centrifuged at 1,300 g for 15 min for plasma separation and stored at -80ºC until nucleic acid extraction was performed.

The sample size calculation was performed to assess the sensitivity and specificity of the test, with 204 being the number of samples needed for the study. Considering that patients are tested for CMV on average 8 times during the first three months of follow-up (according to local data) and considering a 20% loss margin, 30 patients were first planned to be included in the study. However, when observing the low adherence of some patients in consultations and exam collections, 51 patients were included. The inclusion criterion was for patients over 18 years of age, who were referred for kidney transplantation in the hospital and diagnosed with chronic kidney disease. The exclusion criterion was not signing the Informed Consent Form.

As part of the routine hospital care, patients received anti-CMV therapy based on antigenemia results, with a threshold of 10 cells/10^5^ leukocytes - patients presenting lower cell counts but showing symptoms attributable to CMV disease were also put on anti-CMV treatment.

### Data collection

Clinical and demographic data were collected for all patients who entered the study. These variables included underlying diseases, induction therapy following kidney transplantation, regimen of immunosuppression, and CMV serology for both donors and recipients.

### CMV Pp65 antigenemia

CMV antigenemia test was performed using the CMV Brite(tm) Kit (IQ Products, The Netherlands), as part of patients' routine monitoring for CMV infection.

### Quantitative *in-house* qPCR assay

The quantitative in-house qPCR assay was analytically validated in a previous study^6^. Plasma samples used for the study were extracted with Maxwell^®^ 16 Viral Total Nucleic Acid Purification Kit (Promega, USA) following the manufacturer's instructions.

Primers and probes used in this study were those described by Ho and Barry and the sequences are shown in the supplementary material with some modification in the probe design^7^.

The PCR reaction was performed to a final volume of 20 µL using 4 µL of ultrapure water, 3 µL of extracted DNA, 0.4 µM of each primer, 0.25 µM of each probe, 10 µL of GoTaq Probe qPCR Master Mix (Promega, USA) and 0.4 µL of carboxy-X-rhodamine (CXR) in a 1:50 dilution. The thermocycling conditions for the qPCR reactions were: 1 cycle of 2 minutes at 50°C, 2 min at 95°C, followed by 40 cycles of 15 sec at 95°C, and 1 min at 60°C, in a 7500 real time PCR system (Thermo Scientific, USA).

The primary calibration standard used was the 1^st^ WHO International Standard for Human CMV (NCBI code 09/132). Material was prepared as indicated by the manufacturer.

The secondary pattern used in the study was a plasmid synthesized by Applied Biosystems (Thermo Scientific, Brazil) with a sequence of CMV genome (supplementary material) and has been validated using the 1^st^ WHO International Standard for Human CMV (WHOIS), generating a conversion factor for international units. The standard had an initial concentration of 9.65 x 10^10^ copies/mL.

To determine the limit of quantification (LOQ) and conversion factor, two different operators performed the analytical sensitivity tests, on three distinct days. The test consisted in a curve which was amplified in parallel for a base 10 dilution of the primary standard and the secondary standards. The limit of detection (LOD) was determined by the lower point of the curve amplified by 95% of the time diluted in base two, in triplicates. The concentration that consistently amplified 95% of the time was tested again, in triplicates.

The conversion factor was calculated by the median of the division of the CMV concentration (IU/mL) from the primary standard (80% efficiency in extraction) by the average number of copies/mL, for both genes, found in the three days of the test for each of the points of the curve of the secondary pattern. Parameters for qPCR are shown in Figure S1 in the supplementary material. Only results above the limit of quantitation and detection were considered positive.

### Statistical analysis

The comparison between the tests was performed using the Cohen's Kappa coefficient and Spearman's correlation coefficient. Results were interpreted according to Altman *et al.* 8 and Akoglu *et al*.^9^, respectively. Comparison of medians of antigenemia and qPCR results between patients who were asymptomatic and symptomatic was made using the T-test for independent samples. A Receiver Operator Characteristics (ROC) curve analysis was performed to determine the threshold to initiate preemptive therapy. Statistical analyses were performed by SPSS Software (Statistical Package for the Social Sciences), version 18.0.

### Ethical aspects

The ethics committees of the Universidade Federal de Ciências da Saúde de Porto Alegre and the Santa Casa de Misericórdia of Porto Alegre approved the present study, in accordance with the precepts of the Declaration of Helsinki by the following protocol numbers: 1.820.875 and 1.885.683. Written consent was obtained for all patients before entering the study. All experiments were performed in compliance with relevant laws and institutional guidelines and in accordance with the ethical standards of the Declaration of Helsinki.

## Results

From December 2016 to December 2017, 300 kidney transplant procedures were performed in the hospital, from which 51 patients participated in the study. Twenty-one patients were excluded due to poor adherence to the collection of laboratory exams and/or missing consultations. The final study population consisted of 232 plasma samples from 30 patients (average of 7.7 samples per patient, ranging from 5-14). Patient demographic characteristics are presented in Table 1.

**Tabela 1:** Demographic characteristics of patients evaluated in this study

Patients Characteristics	Frequency (%)
Recipient	
Sex	
Male	60
Age (years)	
Median (range)	53.5 (21-71)
Race	
Caucasian	83.3
Cause of ESRD	
Unknown	26.7
Polycystic kidneys	20
Focal segmental glomerulosclerosis	13.3
Type 2 diabetes mellitus	13.3
Type 1 diabetes mellitus	6.7
Systemic lupus erythematosus	6.7
Systemic arterial hypertension	3.3
Berger's disease	3.3
Alport's disease	3.3
Chronic glomerulonephritis	3.3
PRA class I (%)	
0	60
1-49	26.7
50-79	10
≥ 80	3.3
PRA class II (%)	
0	40
1-49	33.3
50-79	23.3
≥ 80	3.3
DSA quantity (%)	
1	8
Induction therapy	
Tacrolimus + Mycophenolate sodium + Steroids	100
Antithymocyte globulin	40
Basilixumab	60
Hemodialysis until 1st week after transplantation	
Yes	40
**Donor**	
Sex	
Male	66.7
Age	
Median (Range)	49.5 (1-70)
**Donor/ Recipient serostatus for CMV infection**	
D+ / R+	53.3
D- / R+	33.3
D+ / R-	6.7
D- / R-	3.3

Legend: D: donor, DSA: donor specific antibody, ESRD: end stage renal disease, HLA: human leucocyte antigen, PRA: panel reactive antibodies, R: recipient and SD: standard deviation.

**Figure 1:**
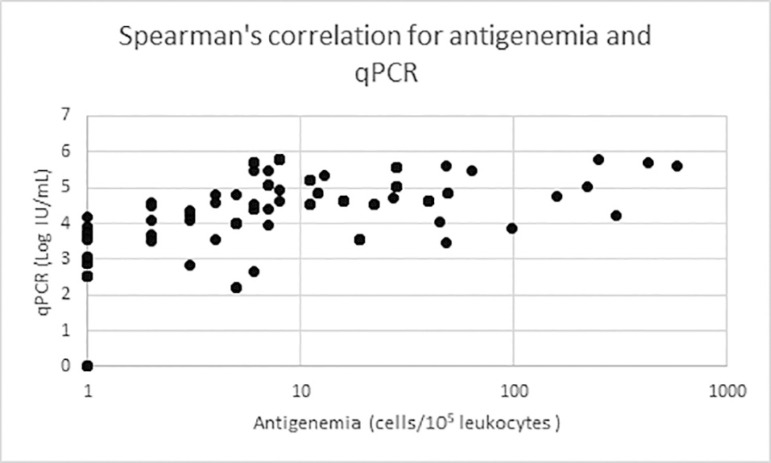
Graphical result for the Spearman's correlation test.

One hundred and two (44.0%) samples were negative for both qPCR and antigenemia. Positive results were observed in 130 (56.0%) samples: 61 (46.9%) were positive for both methods, 68 samples (52.3%) were positive by qPCR only, and 1 sample (0.008%) was only positive by antigenemia. qPCR and antigenemia tests were concordant in 163 samples (70.3%) (Kappa coefficient test=0.435; *p*<0.001, Spearman correlation test=0.663 *p*<0.001). The graph for Spearman's correlation is shown in Figure 1. Of the 69 discordant samples between qPCR and antigenemia, 54 (78.3%) occurred just before (median of 12 days, range, 0-25 days) or soon after (median of 9 days, range, 0-28) antigenemia became positive or negative, respectively. Regarding the 15 samples (21.7%) that were qPCR-positive and antigenemia-negative, the qPCR results varied from Log 2.79 IU/mL to Log 3.97 IU/mL. The only case of positive antigenemia (1 cell/10^5^ leukocytes) with negative qPCR occurred in a patient who presented with DNAemia in previous weeks, and the patient became negative after a few weeks for both antigenemia and qPCR tests. It is important to note that all patients had blood tests, only 4 samples had neutrophil counts below 1000/mm^3^, all of them were negative for both tests. The median leukocyte count was 6845/mm^3^, being 4895/mm^3^ for neutrophiles.

During the study, of the 30 patients included, only five were negative for both tests under comparison. Among the 25 patients with positive tests, 21 (84.0%) had at least one positive result for both tests and four (16.0%) had only qPCR positivity. The Kappa coefficient was 0.636 (*p*<0.001). The median number of days for the first positive result to occur was 50 (range, 24-105 days) for antigenemia and 42 (range, 24-74 days) for qPCR (*p*<0.001). Of these 25 patients, 17 (68.0%) were treated with intravenous ganciclovir for CMV infection or disease, 4 (16.0%) had decreased immunosuppression without the need for antiviral treatment. Four others (16.0%) received no intervention once the antigenemia was negative and the physician were not aware of qPCR results. Of the 25 patients with a positive result, 11 (44.0%) were symptomatic but only 3 (12%) developed CMV disease, and 22 (88.0%) had CMV infection. The symptoms related to CMV were: leucopenia (n=7; 28.0%), thrombocytopenia (n=6; 24.0%), diarrhea (n=3; 12.0%), and oral mucosal lesions (n=1; 4.0%). Pancytopenia was observed in 1 (4.0%) case of CMV disease. A significant difference was found between the median number of cells in patients who were symptomatic and patients who were not: the median was respectively 7.0 cells/10^5^ leukocytes (ranging from 1 to 580 cells/10^5^ leukocytes) and 3.0 cells/10^5^ leukocytes (range, 1-48 cells/10^5^ leukocytes) (*p*=0.021). qPCR results were also significantly different between symptomatic and asymptomatic patients, with median results of 15,539.02 IU/mL (range, 528.66 to 605,059.08 IU/mL) and 3,490.12 IU/mL (range 166.04 to 486,978.25 IU/mL), respectively (*p*<0.001). Among 5 (16.7%) patients who received prophylactic antiviral therapy, all had detectable DNAemia with median results of 9,896.05 IU/mL (range, 528.66 to 605,059.08 IU/mL) but none developed disease. Of the 25 (83.3%) patients on preemptive therapy, 20 (80%) developed CMV DNAemia and 3 (12%) had CMV disease.

Evaluating donors and transplant recipients according to the CMV serology status, of the 30 patients, 16 (53.3%) were D+/R+, and 15 (93.8%) of them presented CMV DNAemia and 2 (12.5%) developed CMV disease. In the D-/R+ group, 90.0% had CMV DNAemia and 1 developed CMV disease. In D+/R- patients, 50.0% had DNAemia. The only patient in the D-/R- group did not present CMV DNAemia.

**Figure 2:**
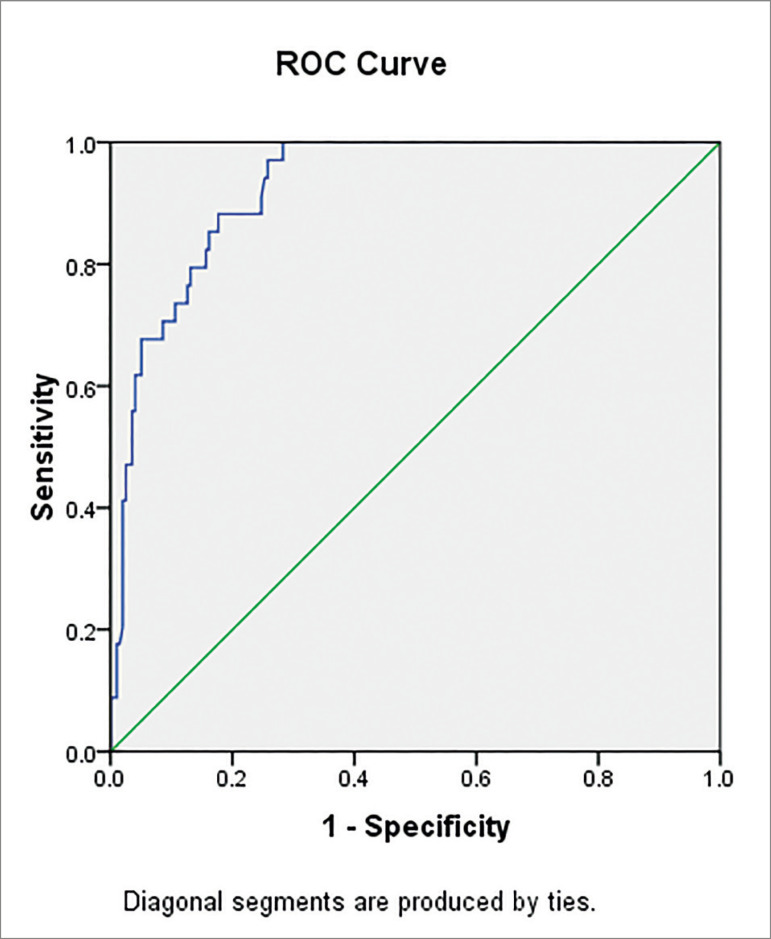
Performance of the in-house qPCR test in the prediction of relevant CMV antigenemia results (i.e., threshold used in the institution to initiate anti-CMV therapy, 10 cells/105 leukocytes), as well as and physicians' decision to initiate anti-CMV therapy. Three thresholds were tested: 2,750 IU/mL (Log 3.44), 3,430 IU/mL (Log 3.54) and 3,650 IU/mL (Log 3.56), resulting in qPCR sensitivity of 100.0, 97.1, and 91.2%, respectively. Specificity for the same thresholds were72.0, 74.2, and 75.3%, respectively. Considering the sensitivity and specificity of the thresholds, the value of 3.430 IU/mL (Log 3.54) was chosen to initiate therapy (AUC 0.92617 ± 0.0185, *p*<0.001). The Kappa correlation coefficient between qPCR and antigenemia was 0.604.

Figure 2 shows the performance of the in-house qPCR test in the prediction of relevant CMV antigenemia results, as well as physicians' decision to initiate anti-CMV therapy. Three different thresholds were tested and the results of sensitivity and specificity for each one are shown in the figure.

## Discussion

Despite advances in the diagnostic field, CMV infection still results in high rates of morbidity and mortality among solid organ transplant recipients^1^. In this prospective cohort of kidney transplant patients, a high infection rate (83.3%) was observed, while CMV disease occurred in 10.0% of patients. A study performed in the same institution in 2004 using CMV antigenemia as a diagnostic tool observed 60.0% of infection and 38.4% of disease^10^. In a study carried out in another hospital in the same city in Brazil, with a composition of patients that was similar to that of this study, the incidence of CMV infection was 53.3%^11^. This cohort was characterized by an elevated seroprevalence of CMV infection in both donors and recipients, and by a limited proportion of patients on universal anti-CMV prophylaxis (16.6% of patients in comparison to 50.0% in the study by Franco *et al*. (2017)^11^^,^ 12 ^,^ 13. Another study conducted in Brazil in a low-risk population of kidney transplant recipients found an incidence rate of 69.6% using antigenemia and qPCR^14^ methodologies, yet a cohort study performed in heart transplant recipients found a rate of 93.3% incidence^15^. The incidence rates found in Brazil are similar to studies in Japan (70.8%)^16^ and India (73.7%)^17^ but differ from countries such as Korea, where the literature shows rates of 30-40%^18^^-^ 21, Finland of 27%^22^ and in the USA, in a pediatric kidney transplant population, a rate of 27% was found.^23^

The comparison between the two diagnostic tests performed in this study showed a concordance between the results of 70.3%, in agreement with previous studies that demonstrated concordances ranging from 66.6-94.3%^11^^,^ 15 ^,^ 18 ^-^ 20 ^,^ 23 ^-^ 25. However, most of these studies were performed before the advent of the WHOIS, as well as before the knowledge of factors related to the presentation of the virus in different biological matrices^5^^,^ 26 ^,^ 27. These factors drastically influence the reproducibility, sensitivity, and specificity of molecular tests. Kamei *et al*. (2016)^25^ found agreement of 87.4% between methodological results using a WHOIS calibrated assay in liver transplant patients^2^. Kappa test revealed a fair agreement between the tests, which was also seen by Franco *et al*. (2017)^11^ *,* Rhee *et al*. (2011)^28^, and Choi *et al*. (2009)^21^. In the studies of Rha *et al*. (2012)^23^ and Kwon *et al*. (2015)^18^, strong concordances were found (0.61 and 0.66). The correlation between the tests was fair when the threshold of Log 3.44 and Log 3.56 were considered for positive results and moderate when Log 3.54 was used as threshold. The agreement between the tests becomes good when evaluated among the patients, similar to previous studies^11^. When performing an analysis to evaluate the correlation between the assays, we found a moderate relationship; this result was also found by Ishii *et al*. (2017)^16^, Kamei *et al*. (2016)^25^ and Rhee *et al*. (2011)^19^. This moderate relationship between tests could be explained by the fact that antigenemia is an operator-dependent semi-quantitative technique and qPCR is a quantitative technique that allows the automation of several steps. In addition, most of the discordant results are explained by the greater sensitivity of the molecular assays when compared to the antigenemia, since the positive results in the qPCR turned positive and negative more than a week before and after the CMV antigenemia test^18^. It was also observed that only 4 samples had neutrophil counts below 1000/mm^3^, which is one of the limitations of analysis for the antigenemia technique^3^, but all of them were negative for both tests, therefore not considered one of the causes of discrepancy between tests. The median number of days for positivity of antigenemia was 50 and for qPCR, 42. This result is similar to that found by David-Neto *et al*. (2014)^29^ in a double-blind study to determine the cut-off point for initiation of treatment by the preemptive strategy in low-risk kidney transplant patients^29^. It is important to note that most of the patients in this study were considered low risk (D+/R+).

After nearly ten years of the launch of the WHOIS, a consensual threshold for treatment of CMV has not yet been defined. The third international consensus on the management of CMV^3^^,^ 30 in patients with solid organ transplants indicates that it is desirable for centers to define their own threshold taking into account the type of assay, type of biological sample, and risk factors of the patients^3^. In order to balance the sensibility and specificity of the threshold, 3,430 IU/mL (Log 3.54) was chosen to initiate the therapy if 10 cells/10^5^ leukocytes on antigenemia and physicians' decision to treat were used as the gold-standards. The sensitivity of the threshold established in this study was very high (97.06%) while specificity was not optimal (74.2%), but it is important to emphasize that most of the results occurred days before or after positive antigenemia results. Previous plasma studies used different threshold values for low-risk patients, one including 3,983 IU/mL (log 3.60 IU/mL), and another 2,750 IU/mL (log 3.44 IU/mL) for low-risk patients, and 1,500 IU/mL (log 3.18 IU/mL) for high-risk patients^31^^,^ 32. Considering the patients in this study with positive qPCR results who had negative antigenemia (4/30), only two reached the threshold point of Log 3.54 - therefore, specificity was 93.3%.

This study has some limitations, the main one being the small number of patients investigated. Twenty-one patients were excluded from the study due to poor adherence to the collection of laboratory exams or missing consultations. Additionally, the study population is composed mostly of low-risk patients, not allowing our threshold values to be generalized to other patient populations. However, we emphasize that this occurred due to the high seroprevalence of CMV infection in this population.

In conclusion, the two CMV diagnostic tests used in this study, qPCR and antigenemia, showed a fair correlation. Recent knowledge on the relevance of viral kinetics allows for the development of increasingly sensitive molecular tests and better evaluation of CMV DNAemia in patients, with positive results ahead of what was previously seen with antigenemia only. However, this high sensitivity requires a careful clinical evaluation of the threshold for the initiation of treatment, in order to avoid unnecessary treatment. Here we demonstrated the optimal threshold value for a novel in-house qPCR in the management of CMV infection in kidney transplant patients, using the WHOIS as a standard. More studies using qPCR calibrated with the WHOIS are needed so that thresholds can be compared in the search for one that can be extrapolated to populations of patients with different risks.

## Supplementary material

The following online material is available for this article: Supplementary Material to "Clinical validation of an in-house quantitative real time PCR assay for cytomegalovirus infection using the 1st WHO International Standard in kidney transplant patients"
