# Supplementary material for: Clinical validation of an in-house quantitative real time PCR assay for cytomegalovirus infection using the 1st WHO International Standard in kidney transplant patients
Source: J Bras Nefrol. 2021 May 10;43(4):530–8. doi: 10.1590/2175-8239-JBN-2020-0214 (PMC8940123; doi:10.1590/2175-8239-JBN-2020-0214)
Supplement: Supplementary file 1 [file 2175-8239-jbn-2020-0214-suppl01.pdf]

Brazilian Journal of Nephrology
https://doi.org/10.1590/2175-8239-JBN-2020-0214

Supplementary Material to “Clinical validation of an in-house quantitative real time PCR assay for cytomegalovirus infection using the 1st WHO International Standard in kidney transplant patients”

The PCR reaction was performed to a final volume of 20 µl using 4 µl of ultrapure water, 3 µl of extracted DNA, 0.4 µM of each primer, 0.25 µM of each probe, 10 µl of GoTaq Probe qPCR Master Mix (Promega, USA) and 0.4 µL of carboxy-X-rhodamine (CXR) in a 1:50 dilution. The thermocycling conditions for the qPCR reactions were: 1 cycle of 2 minutes at 50°C; 2 min at 95°C; followed by 40 cycles of 15 sec at 95°C, and 1 min at 60°C,in a 7500 real time PCR system (Thermo Scientific, BR).

The primary calibration standard used was the 1st WHO International Standard for Human Cytomegalovirus NCBI code 09/132. Material was prepared as indicated by the manufacturer.

The secondary pattern used in the study was a plasmid designed for target both primers and probes sequences. The standard had an initial concentration of 9.65 x 10^{10} copies/mL.

To determine the limit of quantification (LOQ) and conversion factor two different operators performed the analytical sensitivity test on three distinct days. The test consisted in a simplicata curve which was amplified in parallel for a base 10 dilution of the primary standard and the secondary standard. The limit of detection (LOD) was determined by the lower point of the curve amplified by 95% of the time diluted in base two, in triplicate. The concentration that consistently amplified 95% of the time was tested again, in triplicate.

The conversion factor was calculated by the median of the division of the IU/mL amount from the primary standard (80% efficiency in extraction) by the average number of copies/mL, for both genes, found in the three days of the test for each of the points of the curve of the secondary pattern. Parameters for qPCR are shown in Figure S1.

Primers and probes used in this study targeted the genes UL34 and UL80.5. Sequences are shown below.

Table S1. Sequences of primers and probes for genes ul34 and ul80.5.

Genes	Sequences (5'-3')
UL34	F- TGAACTTCATCATCACCACCCGAGACT
	R- CCTTGTACGCTTTGGAAATCGAGCCTG
	P- FAM-CGACGATTCAGTCCTGCGAGCC-QSY
UL 80.5	F- CGGCTAGTGTCGTGTTAGC
	R- CACAAAAATCCGCCGATTCAGATC
	P- VIC-AAGCCGCCGCAGCTTCCCAG-QSY

The sequence of the plasmid used for the standard curve is shown below and was synthesized by Applied Biosystems (Thermo Scientific, BR). Primers and probes are shown in bold for UL34 gene, and in underline for UL80.5 gene:

CCCCACCGCCGTCGTCGTCA**TGAACTTCATCATCACCACCCGAGACT**TCTCCAA**CGACGATTCAGTCCTGCGAGCC**GCCGAGATGCGTGACAACGTGG**CAGGCTCGATTTCCAAAGCGTACAAGG**GCACGGTACGCGCCGAAGGCTTTTTGGCAGGTTCTTCTTCCTGCCCGGCTAGTGTCGTGTTAGCCGCCGCTGCTGCCCAAGCCGCCGCAGCTTCCCAGAGCCCGCCCAAAGACATGGTAGATCTGAATCGGCGGATTTTTGTGGCTGCGCTCAATAAGCTCGA

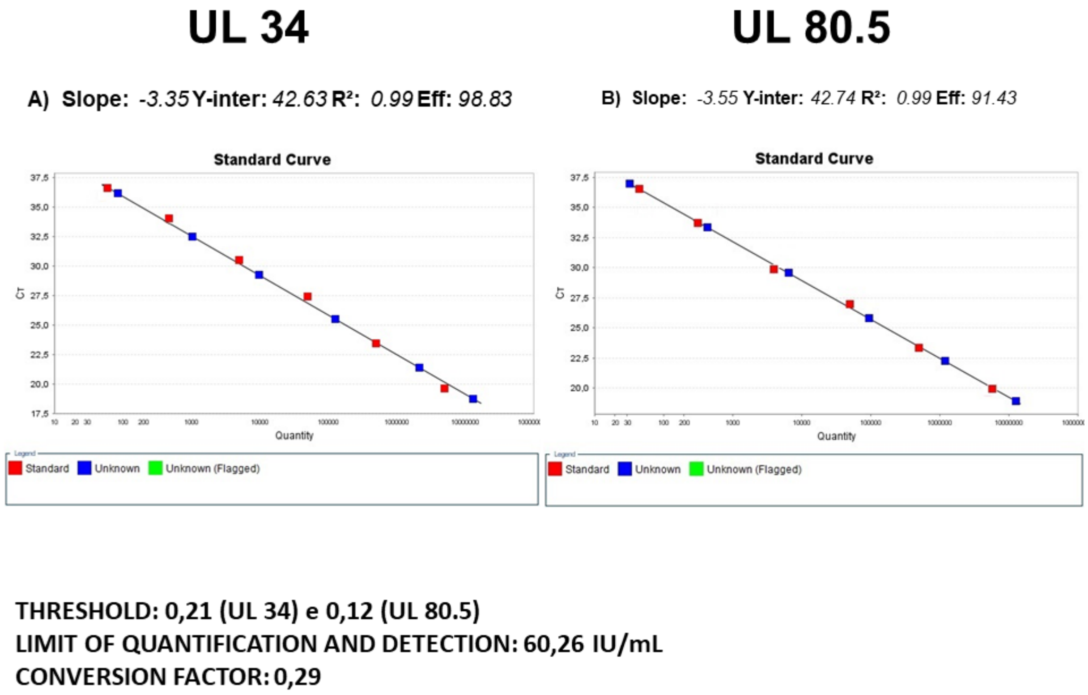

Figure S1 - qPCR Assay Parameters, primary standard in red and secondary standard in blue.
